# PERSONALITY AND COGNITIVE AGING

**DOI:** 10.1093/geroni/igz038.2857

**Published:** 2019-11-08

**Authors:** Eileen K Graham, Stacey B Scott, Avron Spiro

**Affiliations:** 1 Northwestern University, Chicago, Illinois, United States; 2 Stony Brook University, Stony Brook, New York, United States; 3 Boston University, Boston, Massachusetts, United States

## Abstract

Understanding when, how, and for who, cognitive decline occurs is essential to understanding how to optimize quality of life among aging adults. It is well known that there is large variation in cognitive change: the pace and direction of change differs greatly across individuals. Personality traits are one key factor that account for some of these individual differences. Individuals with high levels of certain characteristics are more or less likely to engage in lifestyle behaviors that may put them at greater or less risk of decline over time. The goal of our symposium is to present novel research in this area and discuss the implications for understanding personality and cognitive decline. First, Scott and colleagues will demonstrate a novel approach to personality measurement, and the extent to which there is longitudinal measurement invariance in these measures. This is an important first step in the study of change processes. Second, Terracciano and Sutin will test associations between personality traits and verbal fluency in aging adults, and whether these associations replicate across multiple large panel studies. Third, Graham and colleagues will investigate trajectories of cognitive decline, specifically whether personality is associated with decline both before and after a diagnosis of dementia. Fourth, James and colleagues will discuss the extent to which personality is associated with discordances between dementia diagnosis and neuropathology. All talks will focus on open science, reproducibility, replicability, and generalizability, consistent with GSA’s efforts toward these goals. Discussant Avron Spiro will contextualize these new findings and propose next steps.

